# How Indigenous mothers experience selecting and using early childhood development services to care for their infants

**DOI:** 10.1080/17482631.2019.1601486

**Published:** 2019-04-15

**Authors:** Amy L. Wright, Susan M. Jack, Marilyn Ballantyne, Chelsea Gabel, Rachel Bomberry, Olive Wahoush

**Affiliations:** a Lawrence S. Bloomberg Faculty of Nursing, University of Toronto, Toronto, Canada; b School of Nursing, McMaster University, Hamilton, Canada; c School of Nursing, McMaster University, Holland Bloorview Kids Rehabilitation Hospital, Toronto, Canada; d Indigenous Studies; Department of Health Aging & Society, McMaster University, Hamilton, Canada; e Department of Health Aging & Society, McMaster University, Hamilton, Canada

**Keywords:** Indigenous maternal/child health, qualitative research, community health services, health services research, health promotion, equitable health care, Canada, infants, culturally safe care, early childhood development

## Abstract

**Purpose**: Promoting a child’s healthy growth and development in the first six years of life is critical to their later health and well-being. Indigenous infants experience poorer health outcomes than non-Indigenous infants, yet little is understood about how parents access and use health services to optimize their infants’ growth and development. Exploring the experiences of Indigenous mothers who select and use early childhood development (ECD) services provides important lessons into how best to promote their access and use of health services.

**Methods**: This qualitative interpretive description study was guided by the Two-Eyed Seeing framework and included interviews with 19 Indigenous mothers of infants less than two years of age and 7 providers of ECD services.

**Results**: Mainstream (public) and Indigenous-led health promotion programs both promoted the access and use of services while Indigenous-led programs further demonstrated an ability to provide culturally safe and trauma and violence-informed care.

**Conclusions**: Providers of Indigenous-led services are best suited to deliver culturally safe care for Indigenous mothers and infants. Providers of mainstream services, however, supported by government policies and funding, can better meet the needs of Indigenous mothers and infants by providing cultural safe and trauma and violence-informed care.

Promoting adequate growth and development in the first six years of a child’s life is critical to health and well-being in adulthood (Halseth & Greenwood, 2019; Hertzman, 2000). Self-confidence, language and cognitive skills are achieved during this important period of development and result in readiness for school (Hertzman, 2000). Children who do not develop these skills are more likely to experience lower academic achievement, social, behavioural and mental health issues leading to less economic success as adults (Hertzman, 2000). Health promotion during early childhood is imperative to assist parents in optimizing the healthy growth and development of their children during these critical years. In Canada, early childhood development (ECD) services are provided provincially by public health departments and community services, and commonly consist of parenting classes, programs to promote parent/infant attachment, home visitation services and play groups. Overwhelmingly, participation in these programs is voluntary. Thus, developing a deeper understanding of how parents, specifically mothers, who typically provide for the health needs of their infants, select and the use these programs, will give us insight about the factors that promote their accessibility and use.

This article is part of a larger study exploring the phenomenon of how Indigenous mothers living in the city of Hamilton, Ontario, experience selecting and using health services to meet the health needs of their infants aged less than two years (Wright, 2019). The broader study included 31 participants, comprised of 19 mothers, five primary care providers and seven providers of ECD services. Results presented in this article describe the experiences of mothers specifically related to their use of ECD services to promote the healthy growth and development of their infants. The findings provide transferable strategies to promote the accessibility and use of health services for Indigenous infants and their families in other areas of the health care system.

## Health of Indigenous mothers and infants

Indigenous infants and families in Canada experience difficulties accessing and using health services that are largely associated with the social, political and historical contexts that disproportionately disadvantage Indigenous peoples (Halseth & Greenwood, 2019; Reading, 2015). Literature supports that many Indigenous people experience higher rates of chronic disease, poverty and injury than non-Indigenous people (Greenwood, de Leeuw, Lindsay, & Reading, 2015). Indigenous women experience three times the rate of violence and are more likely to single parent than non-Indigenous women (Amnesty International Canada, 2014; Smylie & Adomako, 2009; Van Herk, Smith, & Andrew, 2011). Indigenous mothers have higher incidences of gestational diabetes and hypertension in pregnancy, and resultant abnormal birth weights that increase their infant’s risk for developmental delay, learning difficulties, hypertension, diabetes and other chronic conditions later in life (Tarlier, Johnson, Browne, & Sheps, 2013). Indigenous infants experience higher rates of birth injury, morbidity and infant mortality than non-Indigenous infants and are at higher risk for admission to hospital (Smylie, Crengle, Freemantle, & Taualii, 2010; Van Herk et al., 2011). It is important to use caution when interpreting the health outcomes of Indigenous people as data are typically aggregated, do not distinguish between the distinct experiences of First Nations, Inuit and Métis groups (Smylie et al., 2011).

The poorer health outcomes experienced by many Indigenous people, particularly women, are a direct result of the lasting impacts of colonization and the implementation of the Indian Act in 1876 (Greenwood et al., 2015; University of British Columbia, 2009). This policy disproportionately disadvantaged Indigenous women; Indian status designations were passed on to children only through Indigenous fathers, women were not allowed to participate in band government, and until 1985, Indigenous women who married non-status men lost their Indian status and its benefits, including the right to live on reserve land (Native Women’s Association of Canada, 2007). This paternalistic hierarchy benefited Indigenous men, and this gender inequality subsequently resulted in poorer health outcomes for many Indigenous women (Barker, 2008).

A recent initiative to collect accurate and representative data for Indigenous people in Canada provided a valid representation of the health status and health service use of First Nations people living in Hamilton, Ontario (Smylie et al., 2011). The findings demonstrated that compared to the general population, First Nations people living in this city had a higher rate of diabetes (15.6% compared to 4.9%) and hypertension (25.8% compared to 19.7%), as well as a larger proportion of individuals who reported fair or poor mental health (36% compared to 10% of those ages 12 years and older living in cities in Ontario) (Smylie et al., 2011; Statistics Canada, 2018). Many First Nations people experienced poverty, with 70% living in Hamilton’s lowest income neighbourhoods, compared to 25% of the non-Indigenous population as a whole (Smylie et al., 2011). Children growing up in economically disadvantaged neighbourhoods have reduced access to parks or green space, and face greater exposure to environmental toxins, air pollution, and noise that negatively impacts brain development, psychological well-being and educational achievement later in life (Moore, McDonald, Carlon, & O’Rourke, 2015). Extreme poverty and inadequate housing represent modes of structural violence, resulting in harm and trauma for those affected, and also leads to food insecurity and other social inequities (Moore et al., 2015; Varcoe, Wathen, Ford-Gilboe, Smye, & Browne, 2016). Indeed, First Nations people living in Hamilton reported high rates of overcrowding (73.7% compared to 3% of Canadians) and difficulties obtaining adequate amounts of food (63% report not buying food in order to meet shelter-related needs; 22% reported sometimes or often not having enough food to eat) (Smylie et al., 2011). Social inequities affecting parents also result in deleterious effects on the health of their children. Parents reported high rates of asthma (14.9%) and chronic ear infections (14%) in their children and 22% reported concerns for their children’s development and for their physical (56.2%), mental (38.2%), and emotional (54%) health (Smylie et al., 2011). From a cultural perspective, 94% of parents felt that traditional events were very or somewhat important to their children’s life (Smylie et al., 2011).

Ongoing and historical trauma can negatively impact the health of Indigenous families. Trauma results when unexpected events occur that surpass an individual’s ability to cope (Manitoba Trauma Information Centre, 2018). Indigenous people uniquely face historical trauma stemming from colonization, and the racist policies which led to the residential school system and the apprehension of thousands of children by child protection services during the Sixties Scoop. Social disadvantage and trauma negatively impact a child’s health, setting the trajectory for reduced health and well-being in adulthood and beyond (Bombay, Matheson, & Anisman, 2009; Varcoe et al., 2016). Despite these risks, Indigenous peoples are resilient; representing the fastest growing ethnic group in Canada, influential leaders and fighting to regain their traditions, cultures and languages (Kirmayer, Dandeneau, Marshall, Phillips, & Williamson, 2011; The Truth and Reconciliation Commission of Canada, 2015).

## Importance of ECD services to development

Access to high-quality ECD services is extremely important to mitigate the effects of social inequity and trauma on the development and subsequent health outcomes of infants. Early childhood development programs support healthy bonding and attachment between parents and infants, provide nutritional and other health-related education, and promote positive parenting and healthy play—all leading to the healthy neurophysiological and psychological development of infants (Gerlach, Browne, & Greenwood, 2017; Halseth & Greenwood, 2019; Moore et al., 2015). Research has demonstrated that by supporting early learning, ECD services are an excellent strategy to promote equity for families facing disadvantage, and promote improved school readiness (Gerlach, Browne, & Suto, 2016; Halseth & Greenwood, 2019; Moore et al., 2015).

How these services are provided is key to promoting access and use by infants and families. Offering this type of education in engaging ways and facilitating communication by using text messaging, for example, effectively involves mothers experiencing poverty and encourages retention (Song et al., 2013). Home visits are another way to engage socially disadvantaged families, as they provide care where families live, and also reduce transportation and child-care related barriers to programming (Dmytryshyn, Jack, Ballantyne, Wahoush, & MacMillan, 2015; Gerlach et al., 2017; Moore et al., 2015). Finally, the provision of culturally-relevant programming for Indigenous infants and families supports resiliency, protecting against mental health issues later in life, and promoting self-esteem and well-being (Halseth & Greenwood, 2019; Priest, Mackean, Davis, Briggs, & Waters, 2012).

### Early childhood development services in Ontario

To promote optimal parenting and to support the healthy growth and development of infants and children, the province of Ontario has invested in a range of health and social services programs. This paper focusses specifically on findings that relate to the experiences of Indigenous mothers selecting and using mainstream programs (Ontario Early Years and Ontario Healthy Babies Healthy Children (HBHC) program) and Indigenous-led programming (Early Years, HBHC and mother-infant classes at the Indigenous Friendship Centre (IFC) in Hamilton) to promote the health of their infants (Ontario Ministry of Children Community and Social Services, 2016, 2018; Ontario Ministry of Education, 2018). Indigenous-led programs are self-governed and provide culturally relevant programming for Indigenous families in their local communities. See Table I: ECD Services in Ontario for a brief description of each.

**Table I.** ECD services in Ontario.

	Mainstream Programs		Indigenous-led Programs		
	Ontario Early Years	Ontario HBHC Program	Early Years	HBHC	Mother-infant classes at IFC
Funding	Ministry of Education	Ministry of Children and Youth Services Delivered by Public Health Ontario	Ministry of Education	Aboriginal Healing and Wellness Strategy	National Association of Friendship Centres
Aims	-Parenting support -Early childhood learning -Parent education on child health and development	-Health screening -Developmental assessments -Healthy mother-infant attachment -Link to community resources and supports -Home visiting	-Same as Ontario Early Years -Indigenous teachings, traditions, ceremonies and resources	-Healthy mother-infant attachment -Healthy infant growth and development -Resources and supports -Family advocacy -Indigenous teachings, ceremonies and resources	-Assist urban-dwelling Indigenous families to be healthy and successful
Eligibility	Parents and their children under six years of age	Pregnant women & families with children under six years of age (typical length of enrollment is one year or less)	Parents and their children under six years of age	Families with at least one child under the age of six years	Families with children
Enrollment	Self-enrollment	a) Screening by a health provider after the birth of an infant b) Self-referral during prenatal or post-natal period	Self-enrollment	a) Identified by HBHC FHVs b) Referral from health provider c) Self-referral	Self-enrollment
Expertise	-Early childhood educators -Affiliated PHNs	-PHNs -Lay-person Family FHVs	-Indigenous early childhood educators -Affiliated PHNs	-Indigenous lay-person FHVs	-Indigenous lay-persons

It is important to note that the Indigenous-led HBHC program differs significantly in three ways from the mainstream HBHC program with whom it is not affiliated despite sharing the same name. First, the Indigenous HBHC program in Hamilton is provided by lay-person Indigenous family home visitors (FHVs) without the addition of PHNs and is supplemented by additional programs available at Hamilton’s IFC. Second, there is no automatic referral process; mothers can self-refer or be referred by health providers or an Indigenous FHV. Third, the Indigenous HBHC program in Ontario is funded through the Aboriginal Healing and Wellness Strategy, a unique approach to the governance and provision of health care for Indigenous people in Ontario established in 1994 (Ontario Ministry of Children Community and Social Services, 2018).

## Methods

This qualitative study used interpretive description (ID) methodology as described by Thorne (2016), and applied the Two-Eyed Seeing framework (Bartlett, Marshall, Marshall, & Iwama, 2015) to integrate both mainstream and Indigenous worldviews throughout the research. A third framework, Andersen’s Behavioral Model and Access to Medical Care (1995), informed the interview guide, and ensured all variables relating to accessing health services were explored. The research was philosophically grounded in constructivism and naturalistic inquiry (Thorne, 2016), which recognizes and values the presence of multiple viewpoints and perspectives. The study was reviewed and approved by three consulting ethics bodies including the Hamilton Integrated Research Ethics Board, the Mohawk College Research Ethics Board and the McMaster University Family Medicine program. A full description of the methodology is published elsewhere (Wright et al., in press).

Briefly, ID is a nursing-derived methodology that appreciates the infinite variation in lived experience that contributes to an individual’s belief of truth and reality (Thorne, 2016). Interpretive description takes a pragmatic approach to inquiry, with the purpose of providing clinicians with practical applications of knowledge to benefit the health of patients and families.

Two-Eyed Seeing was initially developed by two Mi’kmaq Elders to emphasize the need to view the world through the best of both mainstream and Indigenous perspectives (Bartlett et al., 2015). The application of this framework to the research is all-encompassing, including the community-based collaborative approach with Indigenous organizations in Hamilton, the involvement of a Métis advisor and First Nations research assistant to provide Indigenous lived-experience and perspective to the research, as well as a collaborative approach to data analysis. A full description of the application of Two-Eyed Seeing to this research will be available in a future publication.

The Andersen *Behavioral Model and Access to Medical Care* framework was used in the creation of the interview guide, to ensure all variables known to influence access to care were discussed with mothers (1995). The model has been widely tested and validated, and used in research with Indigenous people in Canada and Australia (Andersen, 1995; Nabalamba & Millar, 2007; Trinh & Rubin, 2006; Wallace & Macentee, 2012). Variables influencing access to health care include environmental and population factors, health-related behaviours and the health needs of individuals.

### Sampling techniques

A purposeful sample of Indigenous mothers helped to inform the understanding of how they experience the selection and use of ECD services. Inclusion criteria included self-identifying as Indigenous, living in Hamilton, Ontario and caring for an infant less than two years of age. The age of two years was selected to ensure mothers had experienced using a variety of health services to meet routine health needs, including vaccinations and well-baby checks as well as the usual illnesses experienced by infants in their first two years of life. Purposeful sampling techniques identified mothers who had experienced the phenomenon, and theoretical sampling approaches added further depth to concepts discovered in the data (Patton, 2015). Later in recruitment, snowball sampling was employed to find additional participants who had experienced the phenomenon (Patton, 2015). Mothers were recruited through word of mouth, flyers and with the help of staff at the IFC.

Later, health providers were recruited for interviews to triangulate the data and contribute contextual details to the mothers’ experiences. Inclusion criteria included caring for Indigenous mothers and infants in Hamilton, Ontario. Recruitment strategies included phone and email invitations, along with word of mouth. Data relating to the experiences of primary care health providers are discussed in another publication (Wright et al., in press).

### Data collection

The women in this study shared their experiences during semi-structured, in-depth interviews, used to honour the importance of oral tradition to Indigenous people (The Truth and Reconciliation Commission of Canada, 2015). Interviews lasted approximately 90 minutes and were conducted at a convenient location for the mother, most often their home or at the IFC. During the interview, as the participants described the type of health services accessed as well as their experiences engaging with those services, these relationships were visually documented in the form of an ecomap (Hartman, 1995). In qualitative research, the use of ecomaps has been helpful to minimize power imbalances that can exist between a researcher and participant, as both work together to complete the ecomap (Rempel, Neufeld, & Kushner, 2007; Stewart & Allan, 2013). This technique allowed the researcher to return to areas requiring further exploration, and for mothers to easily determine which health services had been discussed and which still required explanation.

Following the analysis of the individual interview data, a discussion group took place after sharing a meal and socializing. This provided an opportunity for mothers to build relationships with each other, positively contributing to their own health and wellbeing. During the discussion group, mothers had the opportunity to review emerging concepts and ideas and researchers gained further insight and clarity. While not a requirement of ID methodology, member checking was important to validate the emerging findings, as the researcher did not have Indigenous lived-experience. Mothers did not ask for data to be removed and confirmed the presence of themes as identified by the researcher and research assistant. All 19 participant mothers were asked to join the discussion group, and eight attended.

Following interviews with the mothers, providers known to have experience working with Indigenous families were interviewed to further explore contextual issues relating to the mothers’ experiences of using ECD services. Providers completed semi-structured interviews lasting from 30 to 90 minutes, most often taking place at the individual’s place of work or by phone. All interviews and the discussion group were audio-recorded and transcribed verbatim for analysis using NVIVO 12 (QSR International, 2018). Data were secured in a locked office.

### Data analysis

Data analysis was conducted by the researcher and research assistant, initially independently and then together over several months to compare ideas and concepts. Broad coding strategies were used initially so as to not ascribe meaning to the data until fully engaged in the process (Thorne, 2016). Applying the Two-Eyed Seeing framework ensured the analysis incorporated both non-Indigenous and Indigenous perspectives (researcher and research assistant), and in this way the meanings from mothers and Indigenous knowledges were promoted. The resulting thematic summary seeks to bridge both worldviews into an understanding of the phenomenon that has clinical relevance and practical application.

### Integrity

Thorne (2016) defines methodological rigor as integrity that is demonstrated in nine ways. First, the entire research process was congruent with the assumptions of constructivism, which underpins ID and is generally in agreement with Indigenous ways of knowing (Kovach, 2009), demonstrating *epistemological integrity* (Thorne, 2016). Next *representative credibility* was evident through triangulating findings with multiple data sources and literature. *Analytic logic* was ensured by the presence of a supervisory committee and the research assistant, who made certain decisions were logical and congruent with the methodology. Next *interpretive authority*, or evidence of trustworthy interpretations of the data, was established by building authentic relationships with members of the Indigenous community prior to embarking on the research; collaborative data analysis with the First Nations research assistant; and validation of the findings by participant mothers. *Moral defensibility*, referring to the obligation of researchers to conduct research that is necessary and of benefit to others, was demonstrated as the research was conducted to benefit the health of Indigenous mothers and infants in accordance with the Calls to Action by The Truth and Reconciliation Commission of Canada (2015). *Disciplinary relevance* was clear, as the study conformed with nursing aims to help individuals achieve their optimal wellbeing. Finally, the results were presented within the context to which they applied to ensure *contextual awareness* within the limitations of their application; to exercise what Thorne (2016) refers to as *pragmatic obligation* (useable knowledge) and *probable truth*, that what is known in the moment may be disputed and determined as false should new data arise. Collectively, the application of these nine components of integrity as described by Throne (2016) contributed to the study’s strong methodological rigour.

## Results

The results that follow are informed by 19 Indigenous mothers and 7 providers of ECD services. The median age of the mothers was 28 years and one third were first-time mothers. All self-identified as Indigenous; 15 identified as First Nations, two as Métis, and two were unsure of their specific Indigenous culture. Providers of ECD services consisted of PHNs, FHVs and Indigenous staff at the IFC. See Table II Demographic information: Participant mothers for further details including mothers’ involvement with ECD services.

**Table II.** Demographic information: Participant Mothers.

**Variable**	**Category**	**Frequency (%)**
Age	<25 years	5 (26)
	26-30 years	8 (42)
	>31 years	6 (32)
Number of Children	First time moms	5 (26)
	2-5 children	14 (74)
Education	Less than High school	9 (47)
	Completed only high school	3 (16)
	Some College/University	7 (37)
Marital Status	Single/Separated	9 (47)
	Married/Common-law	10 (53)
Indigenous Identity	First Nations	15 (78)
	Métis	2 (11)
	Inuit	0 (0)
	Unknown Indigenous culture	2 (11)
Income	Full-time Employment	7 (37)
	Ontario Works (social assistance)	10 (53)
	Disability Support	2 (10)
Change of address during life of infant	Moved at least once	10 (53)
	Same residence	9 (47)
Regular Health Care Provider	Family physician	17 (90)
	Pediatrician	1 (5)
	None	1 (5)
Involvement with ECD services	Mainstream (public) stream	3 (16)
	Early Years	1 (5)
	HBHC	3 (16)
	Indigenous stream	16 (84)
	Early Years	0 (0)
	HBHC	7 (37)
	IFC	12 (63)
	Both streams	3 (16)
	None	3 (16)

***Note*. N = 19. Adapted from** (Wright et al., in press)

The results are presented first by describing how mothers select the ECD services they use to meet the health needs of their infants. Next, three themes that depict the care provided by both mainstream and Indigenous-led services are described, including: (a) *a comprehensive approach to care;* (b) *facilitating access through home visits*; and (c) *promoting engagement in programming*. Finally, a presentation of the four themes that related explicitly to the strategies used by Indigenous-led programs follows, including: (a) *a wrap-around approach;* (b) *building long-term trusting relationships;* (c) *holistic care*; and (d) *family advocacy*.

### Selection of early childhood development services

Given the number of mainstream and Indigenous-led infant-focused programs available to mothers in the community, we sought to identify which services participants in this study were accessing and using. This is summarized in Table II: Demographic Information: Participant Mothers. The majority of mothers (84%) were using Indigenous-led services. The dominant services used were the mainstream or Indigenous-led HBHC programs and the mother-infant classes at the IFC. Mothers were referred to the mainstream HBHC program by health providers. Following contact by a PHN after the birth of their infants, three mothers were enrolled in the mainstream HBHC program, and two mothers received dual-care by both the mainstream and Indigenous-led HBHC programs. Mothers were enrolled in the Indigenous-led HBHC program if they were previously known to the FHV through their involvement in cultural events or other IFC classes. Neither PHNs or other health providers had referred the mothers to the Indigenous-led HBHC program, such that FHVs were concerned that health providers in the city were unaware of their programs. One shared: “I have never really had [a referral] from a family doctor, and that could be because family doctors don’t know that the program exists”. Mothers who chose to attend the IFC mother-infant classes had learned about these through word of mouth or because they lived in the area.

### Common approaches used by both mainstream and Indigenous-led programs

#### Comprehensive care

Mothers described how providers of both mainstream and Indigenous-led programs took a comprehensive approach to care that assessed and then addressed their specific and unique needs. In particular, mothers described how providers connected them with community resources, tailored health teaching and helped with transportation barriers. Mothers described examples of how FHVs taught them how to promote their infants’ learning through play. One mother explained, “Like last time she came, we made these things in ziploc bags. We put [in] glue and paints and taped it on my floor so he could like squish it around”. Yet another mother shared how her infant’s FHV assisted her with transportation: “[FHV] picked up my daughters for me one time, and [brought] me to [Ontario Works], and to go look for an apartment before I moved here”. Many mothers involved with Indigenous-led programs experienced what they described as an emphasis on providing tangible resources to support families, such as groceries, diapers or bus passes. The comprehensive approach to care by providers in both service streams met the needs of infants and mothers as required, helping some to support their infant’s development, while providing necessary resources such as groceries for others. One Indigenous FHV from the Indigenous-led HBHC program described her approach to meeting the unique needs of mothers and families:

There are always unique needs. I have a client that goes to university and I can’t wait for her to graduate…You know all that she really needs is someone to talk to…You know? Just that little bit of encouragement. Somebody to stand in your corner and be like “you got this”… Some are more emotional. Some need that time [home visit] to feel safe, that they can cry. So just allowing them to be individuals. Allowing everyone to have their journey and that it [HBHC program] is there for them. So I am just there to help out.

A mainstream PHN described how she individualized care through her comprehensive approach with families in the mainstream HBHC program:

It really just depends on the family needs and what their learning needs are. Things like that right? So we focus on healthy growth and development, attachment and positive parenting, but we’ll support the family in other ways too. Like I said, through health teaching and connecting to community resources. Kind of just whatever they need really.

A comprehensive approach supported mothers in their parenting and promoted infant health and development through health teaching, support and resources that were directed where required.

#### Facilitating access through home visiting

Home visiting facilitated mothers’ access to the HBHC program by eliminating barriers to care. The delivery of services through home visiting offered many benefits to mothers, such as allowing FHVs to observe the home environment, enhancing their ability to provide timely education and support, and to be responsive to the unique needs of families. Compared to other services, home visiting allowed the FHV to directly identify infant or maternal safety hazards and provide immediate health teaching. For example, one mother shared: “[FHV] teaches me about how to take care of him properly or keeping the house baby-proof”. Another mother shared that her FHV “would offer to hold baby or take the baby while I would get a shower”. Yet another mother shared how her FHV helped to alleviate her anxiety over feeding her infant: “She came over the first time I fed her because I was really nervous about that. Choking or other hazards. So she was there”.

Providers recognized the advantages to home visiting as well, such as taking resources to the home rather than expecting families to make the sometimes-overwhelming effort to attend events in the community. One Indigenous FHV explained:

I have taken that literature into the home as well. Because if mom has six kids, let’s say, you’re not going to expect her to find a babysitter and come to the centre to sit for two hours. So I would take that literature and bring it to the home. Have a conversation with her based on that.

The same FHV described how home visiting also provided a type of social network to families who were otherwise isolated from friends and family:

Just talking so she has someone to bounce ideas off of and say, “This is what I’m doing”. Sort of like a validation. Some parents don’t have anyone to come over and have tea, so we will bring mom a tea. Just an extra pair of hands and eyes so she can get things done…And that is all some people need is for someone to come over and hold the baby for half an hour or forty-five minutes. Even while they go and have a shower. An uninterrupted shower.

Facilitating access to services through the provision of home visiting broke down barriers to accessing care and allowed providers to better meet the unique needs of Indigenous families; by being in the home environment, providers could better identify areas for health promotion and support.

#### Promoting engagement in programming

Providers in both mainstream and Indigenous-led HBHC programs promoted the engagement and ongoing participation of mothers in their programs by using a range of strategies. These included the use of text-messaging and incentives such as meals, and needed household items, among others. Mothers participating in the Indigenous-led HBHC program consistently described using text-messaging to ask their FHV questions related to caring for the health of their infants, such as advice on how to soothe a crying infant or how to tend to a fever. The use of text-messaging allowed mothers to receive timely answers to their questions, lessening anxiety and improving their confidence as mothers. In some cases, mothers began to assume that their FHV could immediately address any urgent health need that arose. One mother shared her assumption that her FHV would always be available to drive her to the hospital when needed:

Like say if she has a very, very high temperature right now and I tried everything. I would call [FHV] to be honest…I can either ask her where she is or give her a text and she will come over immediately or she will drive me to the hospital.

The use of text-messaging was helpful for providers to confirm appointment times and to notify mothers if they were running behind schedule. One of the mainstream HBHC PHNs shared her experiences of using text-messaging to engage mothers:

A lot of our moms, like you can be in the middle of a feeding or changing, and I am calling them. Like honestly who feels like listening to a voicemail and calling them back? We don’t do that anymore, right?.. I can text them back when I have that minute. It makes it so much easier…We don’t have many missed visits because we can remind them the day before.

Despite these benefits, text-messaging also increased the health provider’s workload beyond the regular business day. The previous HBHC PHN shared her concerns about the use of text-messaging:

We are supposed to text in office hours…For some of us though, our phone is also our personal phone…or you see a message that should be replied to…if they text saying the baby is not latching and ethically we have to reply to that. We are not going to wait until business hours. For me, I would do that anyways.

Likewise, an Indigenous FHV shared how she feels obligated to support her clients outside of working hours because she empathizes with their struggles:

The latest someone called me was midnight, but I was up…I just do it because they need it…I was 17 when I had my boy, and it was a struggle. I had no supports…I was young and naïve, and whatever…I could have used them [FHV] if I knew, right? If someone would have told me. But no. So now that is who I am.

Next, Indigenous providers incentivized mothers attending their programs by offering resources that met their needs, such as meals; making crafts that were also needed as household items for their infants—making a blanket or moccasins for example; socializing with other mothers; or the provision of much-needed baby items and/or bus tickets. An Indigenous staff member from the IFC shared how she addressed the high rate of food insecurity affecting the people she serves:

On Fridays I do [a meal program]. That is basically the biggest part, is food security. We do that on a Friday, because during the week, participants can come out and get meals at all of our programs. But on a Friday, the centre closes, so a lot have to go through making it through the weekend because they don’t have enough food. I like to offer that program, so we at least know they have a meal like just for the weekend anyway.

Collectively, the provision of comprehensive care, facilitating access through home visiting and the use of text-messaging and incentives encouraged the recruitment and retention of mothers to programs, by meeting the unique needs of each family.

### Strategies unique to Indigenous-led programming

Providers of Indigenous-led programming demonstrated an awareness of the unique contextual factors affecting urban-dwelling Indigenous families beyond that demonstrated by mainstream providers and used strategies to enhance access and promote the use of their services.

#### A wrap-around approach

Mothers described multiple ways that Indigenous-led programs used a wrap-around approach to meet not only the physical health needs of their infants, but also address the numerous social determinants of health known to contribute to inequity. For example, the IFC promoted education for at-risk youth and young adults by providing an on-site high-school program. Employment needs were addressed through providing free access to internet and employment counsellors. Additional services targeted alcohol and substance abuse while educating and supporting families impacted by fetal alcohol spectrum disorder. One mother described how the multiple services available at the IFC helped her complete her high school diploma:

When I try to get schoolwork done…in the front there are computers there. So I actually did some today while she fell asleep. I got on there and got a couple of things done. It’s a good thing because it’s free, like you don’t have to sign in and stuff.

A FHV from the Indigenous-led HBHC program described her own past involvement with the HBHC program and how its wrap-around approach contributed to her success in life:

When the other woman came in [Indigenous FHV], she focused on basic needs with me. Are you comfortable being a mom?…So it was more like real stuff…like tangible things. Like what are you going to do with the rest of your life?..So I am her success story…I just started going to school and I finished college.

Collectively, Indigenous-led programs and services prioritized addressing a wide range of social inequities experienced by Indigenous families in Hamilton, in order to support and strengthen families, while also holistically caring for their health and well-being.

#### Building long-term trusting relationships

Secondly, the Indigenous-led programs aimed to build long-term and trusting relationships with mothers, infants and families. A significant contributing factor was the longer length of enrollment in the Indigenous-led HBHC program—families consistently remained enrolled in the program so long as a child less than six years of age was in the home. The length of enrollment for families in the mainstream HBHC program was more typically a year or less. Several Indigenous providers shared examples of how their relationships with mothers and families become so strong, they began to feel like family. This contributed to the important goal of breaking down power inequities. An Indigenous provider from the IFC shared her perspective on the benefits of building strong relationships with the Indigenous families with whom she worked:

I find that the biggest thing for us is being able to open up with them and be compassionate. I don’t know, it just feels more like we are a family with them, rather than someone that is above them. So we are kind of on their level, and there to help them kind of thing.

Another staff member from the IFC shared how a relationship with families helped her to better meet the needs of Indigenous mothers and infants:

I try to be more like their auntie, so they can trust me and come for help if they need something. And not try to hide certain stuff or not tell me certain things. I want them to feel they can. So we can really help them, and find out what they need.

In addition to building trusting relationships between health providers and families, Indigenous-led programs also aimed to facilitate mothers building relationships with others. This was accomplished through programs that brought mothers together, often to create a craft or make a meal. One participant shared how a mothers-only program allowed her to take a break with peers:

…we can just vent because there are no kids allowed for that program. It is nice for the break. We do like crafts and stuff, and traditional beading, and we made a drum and stuff.

New relationships formed at the IFC while infants were young continued to grow, as programs were offered to all age groups. Many mothers described enjoying events with their infant and older children. One mother described how the IFC’s emphasis on forming relationships has strengthened her family:

…before, I had all these problems. I put the kids in school, go home, daycare, then go home and sit there all day…it was like we were always at home. We weren’t doing anything, and the kids would be off the wall…Bringing them here has made a difference between me, [husband] and all the kids. So we’re actually getting closer instead of away from each other.

Building trusting relationships was essential to making mothers feel comfortable sharing their needs with providers, who were then better able to address them. Relationships between mothers, peers and other families were equally important to supporting their parenting journeys through socializing, learning from experienced parents, and promoting family cohesiveness.

### Holistic approach

Mothers consistently described the ability of Indigenous-led programs to provide holistic care; meeting a range of health needs other than merely physical, by assisting emotional connections and attachment between mothers and infants and promoting infant development through play activities and crafts. Providers in the Indigenous-led programs demonstrated a unique ability to care for the cultural and spiritual needs of infants, something mothers did not perceive mainstream services to offer. Mothers spoke of participating in and learning about traditions and ceremonies and that providers took part as well. One mother shared an example of how participating in traditions with her son would help him feel a sense of belonging as he gets older:

He gets to see that he is not different. Because a lot of kids he knows don’t have long hair, and don’t play on drums and all that other stuff…It is good to see his nationality.

An Indigenous staff member from the IFC explained that providing opportunities for urban families to learn about their Indigenous culture was important because they had few opportunities to engage in traditions and ceremonies off-reserve. Another Indigenous staff member emphasized the importance of Indigenous identity to understanding one’s path in life:

All of the classes [at the IFC] have traditional teachings with them… to find out who they are and where they come from. Like what cultural stuff they know, because a lot of them really don’t know a lot. I think if you know who you are and where you come from, and your background, it makes everything else easier. Because you start to realize why you do things…what their family was like and [maybe] what they would like to see different.

Meeting the cultural and spiritual needs of their infants was extremely important to mothers, often a primary reason for attending Indigenous-led programs. The use of traditional medicine in ceremony and smudging was key in meeting the spiritual needs of infants. Some mothers shared their difficulty in obtaining traditional medicines in the city, and that they often relied on friends who lived on-reserve, staff at the IFC or their Indigenous FHV to get these medicines for them. Indigenous providers recognized how this barrier affected mothers living off-reserve and tried to make traditional medicines available. One Indigenous staff member from the IFC described her approach to providing mothers with traditional medicines:

I talk about medicines as well and I tell them where they can go to get their medicines. But for some of them it is hard…so I do have a couple medicines that I can give them, and they want to try it…Like if they sound interested and they can’t get them, then I can get the medicines for them…when I get them, I go to the reserve.

Family home visitors and staff in Indigenous-led programs demonstrated a holistic approach to health promotion unique to the approach taken by providers in mainstream programs. This holistic approach was important for mothers wishing to connect with their Indigenous identity and supported their imparting these lessons on their infants.

#### Family advocacy

While both the mainstream and Indigenous-led programs advocated for families involved with child protection services, mothers described how providers in the Indigenous-led programs made this a priority. Many mothers were involved with child protection services (e.g. Children’s Aid Society) and several were working tirelessly to regain custody of their children. One mother shared how her Indigenous FHV went above and beyond, by supporting her at meetings with child protection services and fostering confidence in her ability to maintain custody of her son:

The native Healthy Babies worker, she has been working with me with CAS [Children’s Aid Society] and that was my biggest challenge. Where I made sure he stayed in my care because I was dealing with addiction and then I was on methadone. But she has helped a lot. She has come to everything, like even if I called her and said “Okay, well they [child protection services] want to meet tomorrow at 11”. She would drop everything and come…any questions I had, really she helped with, and just like everyday life. She has done a lot of referrals and stuff like that for me.

Another mother emphasized how the support she received from staff at the IFC was

instrumental in helping her and her husband regain custody of their children:

They do help a lot. It is like they bring the family together and you do family things together and stuff like that. If you ever need help with anything, they help you…it is so amazing what these people can do for someone…they bent over backwards to help us try to get the kids back…they were coming to court, they were supporting us…I am so happy that I actually found this place.

Understanding the context in which the mothers and infants in their programs lived was key to informing the approach to family advocacy taken by Indigenous providers. One Indigenous FHV shared her perspective of how this approach was proving successful for Indigenous families in her care:

Budgeting and making sure they’re confident when speaking to the [child protection services] worker. They’re scared, and they feel like they have to do things… Like [child protection services] want you to go to parenting, they want you to go to mainstream. You can come to us. Like you can make those suggestions and I make sure those are followed through… [child protection services] says you need to do this, but don’t give them any guidelines because [mothers] have no clue where to go. They don’t even know who to call…it’s basically setting them up to fail as well, right? Those are my goals…the success is more having their file closed from [child protection services], and there has been a lot more in the past two years than in my whole time here.

Mothers involved with child protection services found the support and advocacy of their Indigenous providers key to feeling confident as parents and empowered to meet the requirements of child protection services to keep their children in their care and their families intact. The contextual awareness demonstrated by Indigenous providers and the approach of Indigenous-led services facilitated their ability to provide holistic care which strengthened the family unit and set them up for success.

## Discussion

The results of this study contribute to a growing body of research on Indigenous early childhood health and well-being, and advance a new understanding of how urban-dwelling Indigenous mothers select and use health services to meet the health needs of their infants (Gerlach et al., 2017; Gerlach, Browne, Sinha, & Elliott, 2017; Gerlach et al., 2016; Halseth & Greenwood, 2019; Wright, Wahoush, Ballantyne, Gabel, & Jack, 2018). Mothers involved in both mainstream and Indigenous-led services described how a comprehensive approach and the use of incentives and home visiting facilitated their access to services and promoted their engagement. Despite mothers’ reports of excellent care by mainstream PHNs and FHVs, this study demonstrates that Indigenous-led programs are optimally positioned to promote access and use of ECD services for Indigenous mothers and infants. Providers of Indigenous-led programs demonstrated this by recognizing the unique contextual factors influencing Indigenous mothers and infants and their socially responsive approach to care which included (a) building trusting relationships, (b) meeting social inequities, (c) recognizing and mitigating the impacts of historical, intergenerational and ongoing trauma—particularly through family advocacy, and (d) by providing for the holistic needs of families including cultural and spiritual needs.

Building trusting relationships between providers and mothers is pivotal to promoting engagement in ECD services, especially when child protection services are involved (Gerlach et al., 2017). Despite this, providers struggle to build relationships with Indigenous mothers who commonly mistrust health providers for fear of child apprehension or other negative consequences if they were to be honest about their needs (Munns et al., 2016). In a study of Indigenous infant development programs in British Columbia, FHVs reported spending months to years to develop the level of trust necessary to engage parents in programs and effectively meet their needs (Gerlach et al., 2017). Similar findings were demonstrated in a study in Australia, where FHVs invested in building relationships before they could enter the home or engage in health promotion strategies (Munns et al., 2016). The providers from Indigenous-led programs in this study built long-term relationships with families, much longer than was typical in other mainstream services, which facilitated honesty, and led to their more effectively meeting the needs of families.

Indigenous-led programs met a range of social inequities through a wrap-around approach. Providing numerous services within the same IFC centre, including employment, addictions counselling, education, cultural events, family advocacy and others, has been promoted in the literature as a way to reduce inequities facing children and families (Gerlach et al., 2017, 2016; Moore et al., 2015). In contrast, caring for mothers and infants in siloed approaches causes additional stress for providers who find it difficult to facilitate change in the absence of integrated services (Munns et al., 2016). One poignant example of how Indigenous providers demonstrated contextual awareness of their clients’ social inequities was through their recognition of food insecurity. In response, FHVs and staff at the IFC prioritized supplying groceries and meals to families throughout their programming to meet this need.

Family advocacy was another extremely important way Indigenous providers demonstrated contextual awareness of the impact of colonization, the residential school system and the Sixties Scoop, with the resulting over-representation of Indigenous children involved with child protection services (The Truth and Reconciliation Commission of Canada, 2015). Understanding the impact of social inequity on one’s ability to parent, Indigenous providers sought to keep families intact by addressing social inequities and advocating to child protection services for the inclusion of culturally appropriate interventions for their clients. In a study in British Columbia, Indigenous providers used a similar approach to help parents to identify and work on their limitations to regain custody of their children (Gerlach et al., 2017). Likewise, the Indigenous-led programs in this study demonstrated a strengths-based and proactive approach to the support of families that provided them with opportunities to become successful parents.

Indigenous providers were better situated to meet the holistic health needs of Indigenous mothers and infants, which uniquely included the cultural and spiritual needs of infants. In the broader study, mothers did not describe that their infants’ cultural and spiritual needs were met by mainstream health providers or other health providers in primary and acute care settings (Wright et al., in press). The exceptional ability and mandate of Indigenous-led programs to meet the cultural and spiritual health needs of infants may be what attracted mothers to use these specific services in the first place. Having Indigenous lived experience allowed an intimate knowledge of the meaning of traditional teachings, ceremonies, culture and spirituality as it relates to Indigenous people. Indigenous providers were able to thread these values and beliefs through their culturally relevant programming. Mothers described how Indigenous FHVs integrated traditional teaching and ceremonies in the home, by health teaching and making crafts, like beading and creating dreamcatchers and infant moccasins. Mothers also shared how traditional teaching that was incorporated into every class at the IFC, the availability of on-site Elders, and cultural events such as drumming and dancing were important ways to meet their own cultural and spiritual needs and those of their infants, particularly when such access was often difficult in an urban setting. Other reports in the literature suggest that facilitating cultural connections is important for those living in cities, where Indigenous families may experience cultural and spiritual disconnect through the impact of colonization, and where cultural connections can contribute to building confidence and self-esteem in children as they mature (Gerlach et al., 2016; Priest et al., 2012). Providers of Indigenous-led programming in Hamilton evidently understand this dilemma and work diligently to support the cultural and spiritual well-being of families.

The approach to care taken by providers of Indigenous-led programs is an excellent illustration of culturally safe and trauma and violence-informed care (TVIC). The foundation of culturally safe care, initially defined by Maori nurses in New Zealand, is an awareness of the differences that exist between people and of ensuring that caring for another is not negatively influenced by differing values and beliefs (Papps & Ramsden, 1996). When providing culturally safe care for Indigenous people, health providers require an understanding of the unique contextual factors that impact health and well-being, including colonization, the residential school system, resulting trauma as well as other social inequities (The Truth and Reconciliation Commission of Canada, 2015). Indigenous-led services such as those in this study, are optimally positioned to provide culturally safe care for Indigenous people as Indigenous providers have lived-experience and thus a visceral understanding of the impact of colonization and resulting systemic racism and structural trauma that affect their clients (The Truth and Reconciliation Commission of Canada, 2015). Providers taking a TVIC approach to care have an awareness of the potential of their clients experiencing further harm and/or trauma due to the influence of differing values and beliefs as well as policies and societal structures on their provision of care (Varcoe et al., 2016). Certainly, the mothers in this study confirmed the ability of their infants’ providers from Indigenous-led programs to provide culturally safe and TVIC through an emphasis on building long-term trusting relationships, considering the context of their lives, promoting intact families and through providing holistic care that met their infant’s spiritual health needs. As supported by findings in the literature, ECD services for Indigenous infants and children are most effective when they are developed and led by the Indigenous community itself (Smylie et al., 2016).

### Implications

The lessons learned from ECD services, particularly those that are Indigenous-led, have important implications for practice, policy and future research.

#### Practice

First, health providers can provide culturally safe and TVIC by first reflecting on their own values and beliefs and how these may impact the care they provide (Papps & Ramsden, 1996; Varcoe et al., 2016). Building trusting relationships enables effective care but is often difficult to achieve in today’s health care system in which health providers are increasingly overworked and short-staffed. Next, providers in mainstream programs can acknowledge the importance of meeting the cultural and spiritual needs of infants, and work to link families with appropriate resources in the city to meet these needs. First, however, a better awareness of the availability of local Indigenous-led resources is necessary in Hamilton, which requires more effective partnering and information sharing with Indigenous organizations and services. Finally, PHNs can consider a dual approach to the care of Indigenous mothers and infants by collaborating with Indigenous-led programs. For instance, PHNs and FHVs in the mainstream HBHC program can work together with FHVs from the Indigenous-led HBHC program to better meet the cultural and spiritual needs of Indigenous mothers and infants.

#### Health policy

Second, changes to policy at government and organizational levels are required to enable nursing practice changes. Adequate funding for organizations and educational institutions is required to support training for health providers in the foundations and applications of cultural safety and TVIC approaches to clinical practice. Additionally, strategic partnerships between primary, acute and community care services are required to promote effective coordination of services, enabling a wrap-around approach to care that has been demonstrated to more effectively meet social inequities than siloed approaches (Harrop, Urban Aboriginal Knowledge Network, & Atlantic Research Centre (UAKN Atlantic), 2017). Indigenous-led organizations such as the IFC, are best positioned to provide culturally safe care to Indigenous families, and thus require health policy and adequate funding that supports their ability to continue to meet the health needs of Indigenous families in urban settings. In particular, Indigenous-led programs can benefit from policy and funding models that support integrating Indigenous PHNs into services. Indigenous families should be afforded the equal opportunity to benefit from the expertise of PHNs as families in mainstream programs.

#### Research

Finally, future research is required to understand which health promotion interventions are best-suited to meet the health needs of Indigenous infants and their families as well as how to facilitate their access and use. In particular, how Indigenous infants with chronic health conditions access and use ECD services remains unknown. Research is additionally needed to determine how to equip non-Indigenous providers so they too can appropriately care for Indigenous families in urban areas in culturally safe and trauma and violence-informed ways.

### Limitations

This study focused on the perceptions of mothers who accessed and used ECD services to meet the health needs of their infants, therefore the important experiences of mothers who do not use ECD services or who dropped out of programs were not elicited. Additionally, most participant mothers were First Nations, therefore the experiences in this study may not reflect the experiences of all First Nations, Métis and/or Inuit mothers. Similarly, the experiences of fathers or other parents were not elicited in this study and may also have important implications on the access and use of ECD services. Finally, the infants in this study were healthy and generally not experiencing chronic health conditions. Mothers of infants with chronic health conditions, therefore, may experience different barriers to accessing and using ECD services which require further exploration. Although this study may not be all-encompassing—and was not intended to be fully representative—the results still clearly suggest strategies to improve and produce best results in health care access and delivery for Indigenous infants and their families.

## Conclusions

This study provided a unique opportunity to explore and determine the key characteristics of how Indigenous mothers experience accessing and using ECD services to meet the health needs of their infants. Results suggest that holistic care, facilitated by a wrap-around approach and the building of long-term, trusting relationships is an important way to meet the numerous social inequities experienced by some Indigenous mothers and infants. Mainstream providers are encouraged to become trained in the provision of culturally safe and TVIC, especially as these approaches may offer important ways to support the access and use of health services by Indigenous infants and their families in other health care settings.
